# Dietary Acid-Base Balance in Adolescent Sprint Athletes: A Follow-up Study

**DOI:** 10.3390/nu3020200

**Published:** 2011-02-09

**Authors:** Dirk Aerenhouts, Peter Deriemaeker, Marcel Hebbelinck, Peter Clarys

**Affiliations:** Department of Human Biometry and Biomechanics, Vrije Universiteit Brussel, Brussels 1050, Belgium; Email: pderiema@vub.ac.be (P.D.); mhebbel@vub.ac.be (M.H.); pclarys@vub.ac.be (P.C.)

**Keywords:** acid-base balance, adolescents, potential renal acid load, sprint athletes

## Abstract

Sprinters are advised to include additional protein sources in their diet. Basal metabolism and vigorous physical activities generate hydrogen ions that need to be buffered. The present follow-up study estimates the dietary potential renal acid load (PRAL) and net endogenous acid production (NEAP) in adolescent sprint athletes. Seven-day food diaries and anthropometrics of 60 adolescent sprint athletes (mean age at start 14.7 ± 1.9 years) were collected every six months over a three year period. Comparisons were made between athletes with a negative (PRAL(−)) *versus* positive PRAL (PRAL(+)). For the entire sample, mean PRAL values of up to 6 mEq/day were slightly positive despite a relatively high protein intake of around 1.5 g/kg. The NEAP ranging between 42 and 46 mEq/day remained stable during the study period. Athletes with a PRAL(−) (−8 to −10 mEq/day) consumed significantly more fruit and fruit juice than athletes with a PRAL(+) (+9 to 14 mEq/day). Athletes with a PRAL(+) did not consume more meat, fish and poultry than athletes with a PRAL(−). Grains and dairy products were only discriminative between the two groups on one measurement occasion. Lowering the PRAL can be obtained by increasing the consumption of potatoes, fruits, vegetables and vegetable soup.

## 1. Introduction

The maintenance of homeostasis plays a key role in health and sports performance. As such, tissue and blood pH levels, once perturbed, must be returned to normal ranges. Basal metabolism generates organic acids [1] while intensive exercise can cause transient increases of the acid load [2]. For the elimination of a proton surplus, the body has several buffering systems with a crucial role for the lungs and kidneys. A detailed presentation of the different organs and tissues and how they interact in the regulation of acid-base homeostasis was given by Remer [3]. One component that influences acid-base balance in the human body is the composition of the diet [3]. Remer and Manz [4] found a strong relation between the composition of the diet and the urine pH, and introduced the estimated “potential renal acid load” (PRAL) of food items (expressed in milli-equivalents (mEq) H^+^ per 100 g). Fruit and vegetables have a negative PRAL which means that they potentially contribute in buffering hydrogen ions. Foods with high protein and phosphorus content such as meat and cheese have a positive PRAL, and hence potentially increase hydrogen ion production. The estimated diet-dependent net acid production can be calculated as the sum of organic anions from the basal metabolism and the PRAL of all consumed food items [5]. It has been suggested [6,7] that the long–term net acid excretion should not exceed 100–120 mEq/day since this may result in a maximal renal stimulation of acid, decreasing the plasma bicarbonate availability. It is hypothesized that under these conditions the bone structure can release large quantities of minerals to buffer the exuberant acid load [6]. However, the role of the bone structure as a buffer was recently contested by Fenton *et al.* [8].

Growing children and adolescents are dealing with an increased production of organic acids of which the formation is directly related to the body volume [1]. Adolescent sprint athletes need sufficient energy and nutrient intake to meet demands for growth, development and their specific physical activities [9,10]. Acquisition of muscle tissue in adolescent strength athletes requires a dietary protein intake up to twice that of their sedentary peers [11]. The Joint Position Statement of the American College of Sports Medicine, the American Dietetic Association and the Dietitians of Canada [12] advises a daily protein intake between 1.2 and 1.7 g/kg body mass for adult strength athletes. Literature on protein requirements for adolescent sprint or strength athletes is scarce. Boisseau *et al*. [13] conducted a nitrogen balance study on a group of adolescent soccer players and reported a protein need of 1.4 g/kg·day. Daily recovery and fuel needs for athletes with a moderate volume exercise program requires a carbohydrate intake of 5 to 7 g/kg·body mass [14]. Carbohydrate sources are preferably rich in starch such as rice, pasta, bread and potatoes with only potatoes having a negative PRAL value. Therefore an increase of the dietary PRAL estimation can be expected when attempting a high carbohydrate intake through additional consumption of PRAL increasing (full) grain products such as bread, pasta and rice.

Sprinting is a short burst of activity of high intensity relying primarily on alactic and lactic anaerobic energy systems and in case of repeated sprint exercise, aerobic metabolism also becomes involved [15,16,17]. Especially during longer sprints or when rest periods between repetitions are too short to allow complete recovery, sprint athletes have to deal with high amounts of protons causing metabolic acidosis [2]. Oral ingestion of buffering agents such as sodium bicarbonate or sodium citrate has shown to be an ergogenic aid during high intensity exercise preventing decrease of the blood pH [18].

No information exists on the long–term effects of regular strenuous exercise combined with a diet high in protein in relation to the acid load and stress on the buffering systems of the body. Sufficient fruit and vegetable consumption seems to be indispensible to prevent the diet from being an additional stressor of the buffering system in the sprinters’ body.

It is the aim of this study to evaluate the estimated dietary PRAL in relation to protein and carbohydrate intake, and consumption of different foods in adolescent sprint athletes. 

## 2. Experimental Section

### 2.1. Subjects

Based on Flemish Athletics League rankings of 60 m indoor to 400 m outdoor sprint and hurdles disciplines, 120 athletes aged 12 to 18 years were selected and invited to participate in a 3 year follow-up study on sprint start performance, physical development and nutrition. All athletes were involved in regular sprint training and competition for at least 2 years. Due to logistical limitations, only 60 of the 76 responders were retained (29 girls and 31 boys, aged 14.8 ± 1.6 years and 14.7 ± 1.9 years, respectively). Participating athletes and their parents were given detailed information about the study. In accordance with the university’s ethical committee they were asked to give their written informed consent.

### 2.2. Anthropometrics

Standing height was measured to the nearest 0.1 cm using a wall-mounted stadiometer. Body mass was measured with the TANITA-TBF 410, accurate up to 100 g. 

### 2.3. Food Intake

Subjects completed a seven-day food diary in both the spring and autumn period of 2006, 2007 and 2008 in order to estimate mean daily intake of energy, nutrients and foods. The subjects were clearly instructed to maintain their normal eating pattern and to report all foods as accurately as possible considering preparation, composition, time of the day eaten and portion size. For the latter, they were asked to weigh the items, using their personal weighing scale. When not feasible, household measures were given to make an estimate of the portion size [11]. Diaries were analyzed by one and the same investigator using BINS 3.0 software based on the Belgian food data bank [19]. 

The following formula was used to estimate the net endogenous acid production (NEAP) [4,5]:


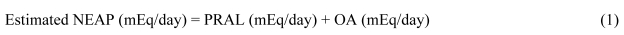


whereby PRAL denotes estimated dietary potential renal acid load and OA denotes estimated urinary organic anions, with the 2 components calculated as follows:


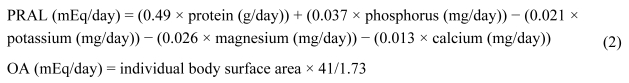


Individual body surface area was calculated according to the formula proposed by Du Bois and Du Bois [20]. Based on the dietary PRAL, for each occasion the participants were divided into a negative PRAL group (PRAL(−), PRAL < 0) and a positive PRAL group (PRAL(+), PRAL ≥ 0). Intakes of meat, fish and poultry, dairy products (milk, yoghurt, cheese, eggs), vegetable and vegetable soup (at least 25% vegetable content), fruit and fruit juices, grain products (bread, rice, pasta) and potatoes (boiled, mashed, fried) were analyzed and compared between the PRAL(−) and PRAL(+) group. 

Basal energy expenditure (BEE) was calculated with the Institute of Medicine equation [21].

The ratio between estimated total energy intake and BEE was used as a tool for detecting underreporting. Since none of the participants followed a calorie restriction diet, the cut off value of 1.1, as suggested by Goldberg *et al.* [22], was used to detect under-reporters. Under-reporters, subjects declaring to have incomplete recordings and subjects reporting less than 7 days, were excluded from the analysis.

### 2.4. Statistics

Statistical analysis was performed with SPSS 17.0. The Kolmogorov-Smirnov test was used to test for normal distribution of the data. To detect variations over time, a repeated measurement ANOVA followed by a paired *t*-test and Bonferroni corrections were applied. In the case of non-parametric data, the Friedman test followed by a Wilcoxon test was applied. Nutrient intakes were compared with the recommended dietary intakes (RDI) of the Belgian Health Council [11] and those proposed by the Health Conference on Diet and Physical Activity [23] with a one sample *t*-test. On each occasion, a comparison of age, height, body mass and consumption of food items was made between the PRAL(−) and PRAL(+) group using independent *t*-tests or, in case of nonparametric data, using the Mann-Whitney U test. The significance level was set at *p* < 0.05.

## 3. Results

The main reasons for subjects dropping out of the study were: a persisting injury (*n* = 8), loss of interest in competitive sport participation (*n* = 7), inability to complete the food diary on one or more occasions due to practical reasons (*n* = 5) and no longer willing to participate (*n* = 4). Five athletes were excluded due to underreporting or incomplete food diaries. Athletes with all six food diaries included in the analysis did not differ in age, height or weight at the start of the study. 

Mean height and body mass increased continuously during the study period. Estimated protein and carbohydrate intake were stable throughout the study period and within the range of, respectively, 1.2–1.7 g/kg body mass and 5–7 g/kg body mass on each occasion (Table 1). More information on anthropometric characteristics and detailed results on macronutrient intake were published by Aerenhouts *et al.* [24]. Mean intakes of phosphorus, potassium and magnesium were significantly higher than the RDI throughout the study period. Calcium intake was significantly below the RDI on all occasions. Calcium/phosphorus ratios were significantly lower than the minimum ratio of 1.0 and the optimal ratio of 1.3 [11]. Phosphorus and magnesium intake at the start of the study were lower than on the third, fourth, fifth and sixth occasion (phosphorus: *p* = 0.006, 0.004, 0.001 and 0.007, respectively, and magnesium: *p* = 0.001, <0.001, <0.001 and =0.005, respectively). Intakes of potassium and calcium remained stable throughout the study period.

**Table 1:** Age, height, body mass, daily intakes of protein and selected micronutrients and estimated dietary potential renal acid load (PRAL) and net endogenous acid production (NEAP) per occasion (mean ± SD).

	Spring 2006	Autumn 2006	Spring 2007	Autumn 2007	Spring 2008	Autumn 2008	ANOVA
	*n* = 48	*n* = 48	*n* = 49	*n* = 46	*n* = 46	*n* = 38	
Age (years)	14.9 ± 1.6	15.4 ± 1.6	15.8 ± 1.6	16.2 ± 1.6	16.8 ± 1.7	17.2 ± 1.6	< 0.001
Height (cm)	169.3 ± 9.5	170.9 ± 8.7	171.4 ± 8.4	172.1 ± 7.4	173.2 ± 7.2	173.8 ± 7.0	< 0.001
Body mass (kg)	54.6 ± 9.8	56.9 ± 9.4	58.2 ± 9.2	59.2 ± 8.8	60.5 ± 8.6	61.8 ± 8.9	< 0.001
Protein (g)	84 ± 19	86 ± 17	87 ± 17	86 ± 17	89 ± 16	91 ± 17	0.066
Protein (g/kg·bw)	1.6 ± 0.3	1.5 ± 0.3	1.5 ± 0.3	1.5 ± 0.3	1.5 ± 0.3	1.5 ± 0.3	0.518
Carbohydrate (g/kg·bw)	5.9 ± 1.2	5.7 ± 1.1	5.7 ± 1.0	5.5 ± 1.0	5.6 ± 0.9	5.7 ± 1.0	0.134
P (mg)	1399 ± 323 ^a^	1467 ± 352 ^a^	1545 ± 383 ^a^	1519 ± 337 ^a^	1566 ± 333 ^a^	1525 ± 333 ^a^	0.037
	1S	2S	1S	3S	2S	2S	
K (mg)	3335 ± 838 ^a^	3388 ± 800 ^a^	3476 ± 842 ^a^	3474 ± 778 ^a^	3565 ± 766 ^a^	3623 ± 808 ^a^	0.489
	0S	1S	3S	0S	1S	1S	
Mg (mg)	299 ± 77 ^a^	324 ± 78 ^a^	337 ± 88 ^a^	343 ± 87 ^a^	350 ± 84 ^a^	336 ± 74 ^a^	0.002
	3S	8S	10S	8S	7S	3S	
Ca (mg)	835 ± 259 ^b^	874 ± 289 ^b^	971 ± 377 ^b^	921 ± 269 ^b^	912 ± 293 ^b^	867 ± 241 ^b^	0.670
	1S	4S	6S	3S	3S	3S	
Ca/P ratio	0.60 ± 0.14 ^b^	0.59 ± 0.13 ^b^	0.62 ± 0.14 ^b^	0.61 ± 0.12 ^b^	0.58 ± 0.13 ^b^	0.58 ± 0.15 ^b^	0.599
Ca/P ratio + S	0.60 ± 0.14 ^b^	0.60 ± 0.13 ^b^	0.63 ± 0.14 ^b^	0.61 ± 0.12 ^b^	0.58 ± 0.13 ^b^	0.58 ± 0.14 ^b^	
PRAL (mEq)	3.8 ± 12.5	5.9 ± 14.3	4.9 ± 13.9 **	4.8 ± 12.0	5.3 ± 10.6	3.8 ± 11.8	0.852
PRAL + S	3.4 ± 12.3	5.6 ± 14.3	4.5 ± 13.7	4.6 ± 12.1	5.6 ± 11.5	3.7 ± 11.8	0.746
NEAP (mEq)	42.4 ± 13.7	45.4 ± 15.3	44.9 ± 14.6 **	45.2 ± 12.6	46.3 ± 10.5	45.3 ± 12.1	0.193
NEAP + S	42.0 ± 13.5	45.1 ± 15.3	44.5 ± 14.4	45.0 ± 12.6	45.9 ± 10.6	45.2 ± 12.2	0.117

S: number of subjects supplementing this nutrient. Significantly above (^a^) or below (^b^) the recommended dietary intake (RDI) [11] at *p* < 0.001. ** Significantly different from value calculated with supplements (+S) at *p* < 0.01.

Dietary PRAL and NEAP estimations remained stable throughout the study period. The PRAL and NEAP estimations with and without supplements included in the calculation differed significantly on the third occasion (*p* = 0.006).

On each occasion, the whole study group was divided into athletes with a negative PRAL (PRAL(−)), and a positive dietary PRAL (PRAL(+)) (Table 2). NEAP estimations were significantly different between the PRAL(−) and PRAL(+) group throughout (*p* < 0.001). There was no difference in age, height, body mass, protein and carbohydrate intake (both absolute and relative to body mass), nor in the calcium phosphorus ratios. 

Quantities of different foods as consumed by all athletes and consumed by the PRAL(−) and PRAL(+) group are shown in Table 3. 

The number of athletes reaching the recommended intake for fruit (250 g/day [23]) ranged between 10 and 13 while the recommendation for vegetables (300 g/day [23]) was reached by 0 to 2 athletes only. For the group as a whole, soup was consumed more during the autumn than during the spring periods (*p* from 0.007 to < 0.001); whilst for vegetables and soup taken together a lower consumption was reported on the first as compared to the other five occasions (*p* from 0.007 to <0.001). PRAL(−) and PRAL(+) groups consumed equal amounts of soup throughout the study period. For vegetables, only in the autumn of 2006, the PRAL(−) group reported higher vegetable consumption than the PRAL(+) group.

Higher fruit juice consumption was reported by the PRAL(−) group on occasion 1, 3, 4 and 6 whereas they reported a higher fruit consumption on occasion 2, 4 and 5. When fruit and fruit juices are taken together, the PRAL(−) group consumed more than the PRAL(+) group throughout.

As to grain products (rice, pasta, bread), the group as a whole reported a lower consumption at the start of the study as compared to occasions 3 to 6 (*p* from 0.010 to 0.001). The PRAL(−) group reported a lower consumption of grain products in the autumn of 2007 only. The PRAL(−) group reported a higher potato consumption at the start of the study only. 

The group as a whole consumed equal amounts of meat, fish, poultry and dairy products throughout the study. The PRAL(−) group consumed more meat, fish and poultry on occasion 6 and less dairy products on occasion 2 than the PRAL(+) group.

## 4. Discussion and Conclusion

In this study, no direct blood or urine measures of acid-base status were taken. Instead, the net endogenous acid production in these athletes was estimated based on nutrient intakes derived from seven- day food diary analyses and anthropometric characteristics. 

The higher needs for dietary protein and carbohydrate sources for sprint athletes, of which most increase the estimated PRAL, make it difficult for sprint athletes to keep their dietary PRAL low. Considering that sprint athletes are regularly involved in exercise that stresses the buffering systems of the body, a negative, or an estimated PRAL close to zero could be worthwhile in reducing or at least not exacerbating this metabolic stress. The importance of buffering capacity has been demonstrated through the ergogenic effects of buffering agents such as sodium bicarbonate or sodium citrate [18]. 

**Table 2:** Characteristics of the PRAL(−) and PRAL(+) group per occasion (mean±SD).

	Spring 2006	Autumn 2006	Spring 2007	Autumn 2007	Spring 2008	Autumn 2008
PRAL(+)	*n* = 29	*n* = 33	*n* = 31	*n* = 32	*n* = 35	*n* = 27
Age (years)	14.9 ± 1.5	15.4 ± 1.5	15.9 ± 1.7	16.3 ± 1.5	16.7 ± 1.7	17.5 ± 1.7
Height (cm)	169.0 ± 10.8	170.6 ± 9.0	171.4 ± 8.8	171.9 ± 7.7	173.2 ± 7.4	172.9 ± 6.6
Body mass (kg)	54.3 ±10.5	56.8 ± 9.4	58.2 ± 9.2	59.7 ± 8.7	60.4 ± 8.7	61.4 ± 8.2
PRAL (mEq/day)	11.7 ± 9.0	13.5 ± 9.1	12.4 ± 9.7	10.2 ± 9.9	9.7 ± 7.4	9.1 ± 7.5
NEAP (mEq/day)	49.9 ± 12.0	52.8 ± 12.1	52.2 ± 11.7	50.5 ± 11.1	50.5 ± 7.7	50.1 ± 8.2
PRAL(−)	*n* = 19	*n* = 15	*n* = 18	*n* = 14	*n* = 11	*n* = 11
Age (years)	15.0 ± 1.5	15.5 ± 1.7	15.7 ± 1.5	16.2 ± 1.8	17.2 ± 1.3	16.6 ± 1.3
Height (cm)	170.8 ± 6.7	172.5 ± 7.4	172.2 ± 7.2	173.4 ± 5.9	174.5 ± 5.6	177.4 ± 6.2
Body mass (kg)	55.9 ± 8.1	58.2 ± 8.8	59.1 ± 8.7	59.3 ± 8.5	62.3 ± 7.0	64.3 ± 9.5
PRAL (mEq/day)	−7.7 ± 6.5	−9.9 ± 9.1	−8.5 ± 9.6	−7.3 ± 6.0	−8.8 ± 5.5	−9.6 ± 9.8
NEAP (mEq/day)	31.4 ± 7.1 ***	30.1 ± 8.4 ***	31.8 ± 8.9 ***	33.3 ± 5.3 ***	32.8 ± 5.6 ***	33.0 ± 12.0 ***

*** Significantly different from PRAL(+) at *p* < 0.001.

**Table 3:** Daily consumption (g/day) of different food items by all athletes and PRAL(−) and PRAL(+) group per occasion (mean ± SD).

	Spring 2006	Autumn 2006	Spring 2007	Autumn 2007	Spring 2008	Autumn2008	ANOVA
all	*n* = 48	*n* = 48	*n* = 49	*n* = 46	*n* = 46	*n* = 38	
PRAL(+)	*n* = 29	*n* = 33	*n* = 31	*n* = 32	*n* = 35	*n* = 27	
PRAL(−)	*n* = 19	*n* = 15	*n* = 18	*n* = 14	*n* = 11	*n* = 11	
M, F, P	151 ± 51	141 ± 42	136 ± 38	140 ± 43	142 ± 49	156 ± 45	0.208
PRAL(+)	151 ± 54	146 ± 41	130 ± 40	141 ± 45	142 ± 47	147 ± 43	
PRAL(−)	152 ± 46	131 ± 43	146 ± 30	138 ± 38	144 ± 59	180 ± 42 *	
Dairy	321 ± 180	295 ± 193	333 ± 204	278 ± 179	305 ± 199	274 ± 153	0.305
PRAL(+)	339 ± 186	335 ± 199	356 ± 211	300 ± 173	329 ± 212	252 ± 132	
PRAL(−)	294 ± 171	213 ± 156 *	291 ± 190	227 ± 188	230 ± 127	331 ± 191	
V + Soup	149 ± 108	214 ± 123	206 ± 131	224 ± 127	195 ± 140	220 ± 115	<0.001
PRAL(+)	123 ± 98	179 ± 91	194 ± 128	201 ± 109	162 ± 95	212 ± 112	
PRAL(−)	187 ± 114	289 ± 152 *	227 ± 137	276 ± 152	300 ± 205	238 ± 126	
Fruit +juice	344 ± 226	319 ± 249	385 ± 303	362 ± 259	397 ± 274	412 ± 260	0.219
PRAL(+)	253 ± 163	238 ± 151	256 ± 141	269 ± 193	320 ± 180	344 ± 203	
PRAL(−)	481 ± 242 **	498 ± 326 **	607 ± 376 ***	575 ± 272 ***	644 ± 372 *	578 ± 317 *	
Grain	227 ± 86	255 ± 104	258 ± 112	257 ± 94	260 ± 104	275 ± 93	0.005
PRAL(+)	219 ± 71	246 ± 95	266 ± 112	275 ± 100	266 ± 106	288 ± 96	
PRAL(−)	240 ± 105	277 ± 122	245 ± 115	213 ± 65 *	241 ± 99	244 ± 82	
Potatoes	121 ± 87	107 ± 55	94 ± 69	104 ± 65	105 ± 64	103 ± 62	0.625
PRAL(+)	95 ± 33	104 ± 52	83 ± 48	90 ± 50	100 ± 62	94 ± 61	
PRAL(−)	158 ± 123 *	116 ± 62	113 ± 94	137 ± 83	121 ± 71	125 ± 61	

M, F, P: Meat, fish, poultry; V: Vegetables; *, **, *** Significantly different from PRAL(+) at *p* < 0.05, 0.01, 0.001, respectively.

Following the RDI for protein of 1.2–1.7 g/kg·day as formulated by the Joint Position Statement of the American College of Sports Medicine, the American Dietetic Association and the Dietitians of Canada [12], the protein intake of around 1.5 g/kg observed in these sprint athletes was adequate. Carbohydrate intake between 5 to 7 g/kg·day allowed a moderate volume exercise program to be sustained. The relative high protein intake did not result in extreme high dietary PRAL nor NEAP estimations. The NEAP was found to be stable over the three year time period and remained below the proposed cut off of 100 mEq/day [6,7], suggesting that the buffering systems of the body would not be stressed to the proposed limit [3]. The NEAP, however, does not take into account the metabolic stress due to sports activities. On the other hand, it should be mentioned that children and adolescents have a lower anaerobic capacity compared to adults. Hence, during high intensity exercise a lower accumulation of muscle by-products is observed as compared to adults. The latter may be explained by a relative higher oxidative capacity, a higher removal of metabolic by-products and a better acid-base regulation as proposed by Ratel *et al.* [25]. As meat, fish and poultry have a high PRAL, it was somewhat surprising that the PRAL(−) and PRAL(+) groups had comparable intakes, with even a higher intake in the PRAL(−) group on the final occasion. Other foods, such as dairy products, grain products, vegetables and especially fruit and fruit juices, showed to be discriminative between the PRAL(−) and PRAL(+) group. Consumption of dairy products and grain products was higher in the PRAL(+) group but the difference was only significant on one occasion. Fruit and vegetable consumption was below the Belgian recommendation throughout the study. However, consumption of soup and fruit juices with a considerable nutrient density potentially helped to prevent the dietary PRAL becoming too high. Even the PRAL(−) group did not reach the Belgian recommendation for vegetables. Soup consumption appeared to be season dependent as it was higher in the colder autumn periods as compared to the warmer spring periods. If for any reason the consumption of vegetables remains low (e.g., taste, digestibility), it can be interesting to opt for vegetable soup considering its high nutrient density and water content [26].

The use of unsweetened fruit juices, for example in preparing sports drinks, can be very useful in keeping the PRAL low. This is confirmed by the higher quantities of fruit juices consumed by the PRAL(−) group on four of the six occasions, whereas fruit consumption was higher on three occasions in the PRAL(−) group. Still, the PRAL(−) group had a mean daily fruit consumption that reached the Belgian recommendation of 250 g on each occasion in contrast to the PRAL(+) group.

Potatoes (boiled, mashed or fried) as a source of carbohydrate reduce the estimated PRAL, in contrast to other carbohydrate sources such as bread, pasta and rice. Potatoes, pasta or rice are an important component of the daily warm meal in Belgium [26]. Our results show that potatoes are indeed a popular food in these athletes, which is an important PRAL reducing agent in the diet, also in the PRAL(+) group. Alexy *et al.* [27] already reported potatoes as an important PRAL reducing component in the diet of German adolescents. Bread is commonly a part of breakfast as well as one of the other main meals but it adds to a positive estimated PRAL value. Nonetheless, bread consumption should not be restricted as it delivers important amounts of carbohydrate, fiber and micronutrients. 

Intakes of phosphorus, potassium and magnesium were in accordance with the RDI on all occasions in contrast to calcium intake and the calcium to phosphorus ratios. Since calcium in the diet lowers the PRAL, sufficient calcium intake is of importance as it also has a crucial role in pubertal bone mineralization, muscle contraction, nerve conduction and blood clotting [12]. Dairy products as sources of calcium all have a positive estimated PRAL, especially cheese (ranging between 13 and 34 mEq/100 g). Milk and non-cheese products only have a moderate positive PRAL and are therefore more suited sources of calcium. Moreover, a review by Roy [28] shows that skim milk can be a very effective recovery drink after both endurance and strength training. Nonetheless, if dairy products are the main source of calcium, sufficient attention should be paid to PRAL lowering foods such as fruits and vegetables. Consuming calcium fortified foods offers another solution as well as taking a calcium supplement. However, athletes should only take dietary supplements when a food-based solution is not available and after consulting a sports nutrition professional [29].

The subjects in this study have similar dietary PRAL estimations as compared to German children and adolescents as reported by Remer *et al.* [7] and Alexy *et al.* [30]. In the latter study, children with a higher dietary PRAL estimation had significantly less cortical area and bone mineral content. Compared to the athletes in this study, Alexy *et al.* [27] reported higher PRAL estimations (with a rising age trend) on a larger sample of 15 to 18 year old boys.

In conclusion, despite a relatively high protein intake, the estimated mean dietary PRAL was found to be only slightly positive. As indicated by the comparison between the PRAL(−) and the PRAL(+) group, the contribution of potatoes and additional fruit juices and soup to fruit and vegetable consumption appears to be important in lowering the PRAL. There is potential for improvement particularly by increasing fruit and vegetable consumption and in the choice for potatoes over pasta and rice as carbohydrate sources. According to the tentative acid-ash hypothesis, in the context of high-intensity training or competition in which blood pH can temporarily decrease, it is possible that the consumption of high PRAL foods such as meat, cheese and eggs may contribute to lower blood pH. Tests are needed to assess whether the addition of more fruit and vegetables or a bicarbonate salt alter athletic performance. Supplementation with buffering agents such as bicarbonate potentially improves performance but cannot replace PRAL lowering foods in the diet. Information about an optimal dietary composition should contain advice towards suitable food choices for both health and performance. Furthermore, it is important to provide each athlete with individual feedback about his or her diet. Additional research on both immediate and long-term relationships between the dietary PRAL, sports performance and sport injuries may give more insight concerning the relevance of implementing sufficient PRAL lowering foods in an athlete’s diet. 
